# 3-Bromo-*N*′-[(2-meth­oxy­naphthalen-1-yl)methyl­idene]benzohydrazide

**DOI:** 10.1107/S1600536811019349

**Published:** 2011-05-28

**Authors:** He-Bing Li

**Affiliations:** a Department of Chemistry and Life Sciences, Xiangnan University, Chenzhou 423000, People’s Republic of China

## Abstract

The mol­ecule of the title compound, C_19_H_15_BrN_2_O_2_, displays a pseudo-*trans* conformation about the N—N bond [C—N—N=C torsion angle = 164.7 (2)°]. The dihedral angle between the planes of the benzene ring and the naphthyl system is 70.1 (2)°. In the crystal, mol­ecules are linked into *C*(4) chains along the *c* axis by N—H⋯O hydrogen bonds.

## Related literature

For related structures, see: Li (2007*a* ▶,*b* ▶, 2008 ▶); Qiu *et al.* (2006 ▶); Yang & Guo (2006 ▶); Yang (2006 ▶). For bond-length data, see: Allen *et al.* (1987 ▶).
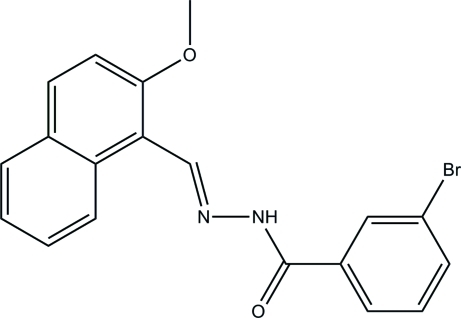


## Experimental

### 

#### Crystal data

C_19_H_15_BrN_2_O_2_
*M* *_r_* = 383.24
Monoclinic, 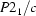

*a* = 12.3562 (11) Å
*b* = 17.0404 (15) Å
*c* = 8.6175 (10) Å
β = 110.155 (2)°
*V* = 1703.3 (3) Å^3^
*Z* = 4
Mo *K*α radiation
μ = 2.43 mm^−1^
*T* = 298 K
0.30 × 0.27 × 0.27 mm

#### Data collection

Bruker SMART CCD diffractometer
Absorption correction: multi-scan (*SADABS*; Sheldrick, 1996 ▶) *T*_min_ = 0.530, *T*_max_ = 0.560
9533 measured reflections
3513 independent reflections
1721 reflections with *I* > 2σ(*I*)
*R*_int_ = 0.080

#### Refinement

*R*[*F* ^2^ > 2σ(*F* ^2^)] = 0.052
*wR*(*F* ^2^) = 0.132
*S* = 1.00
3513 reflections
221 parameters
1 restraint
H atoms treated by a mixture of independent and constrained refinement
Δρ_max_ = 0.30 e Å^−3^
Δρ_min_ = −0.36 e Å^−3^

### 

Data collection: *SMART* (Bruker, 1998 ▶); cell refinement: *SAINT* (Bruker, 1998 ▶); data reduction: *SAINT*; program(s) used to solve structure: *SHELXS97* (Sheldrick, 2008 ▶); program(s) used to refine structure: *SHELXL97* (Sheldrick, 2008 ▶); molecular graphics: *SHELXTL* (Sheldrick, 2008 ▶); software used to prepare material for publication: *SHELXTL*.

## Supplementary Material

Crystal structure: contains datablocks global, I. DOI: 10.1107/S1600536811019349/hb5889sup1.cif

Structure factors: contains datablocks I. DOI: 10.1107/S1600536811019349/hb5889Isup2.hkl

Supplementary material file. DOI: 10.1107/S1600536811019349/hb5889Isup3.cml

Additional supplementary materials:  crystallographic information; 3D view; checkCIF report

## Figures and Tables

**Table 1:** Hydrogen-bond geometry (Å, °)

*D*—H⋯*A*	*D*—H	H⋯*A*	*D*⋯*A*	*D*—H⋯*A*
N2—H2⋯O2^i^	0.90 (1)	2.10 (1)	2.989 (5)	174 (5)

Symmetry code: (i) 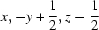.

**Fig. 1.**
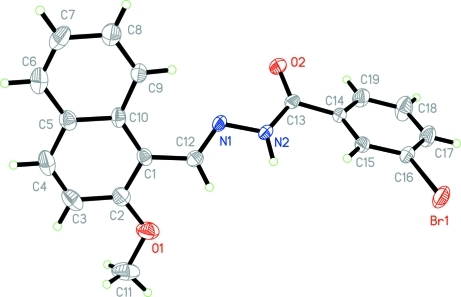
The molecular structure of the title compound, showing 30% probability displacement ellipsoids.

**Fig. 2.**
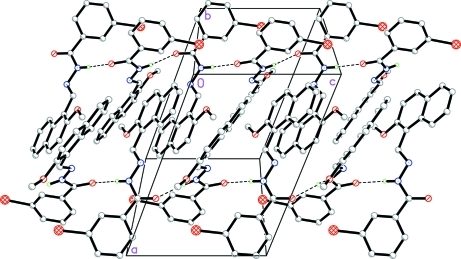
The crystal packing of the title compound. Hydrogen bonds are shown as dashed lines.

## supplementary crystallographic information

### Comment

In the last few years, the author has reported the sturctures of various hydrazone compounds (Li, 2008; Li, 2007*a*,*b*). As an extension of work on the structures of such compounds, the title new hydrazone compound is reported.

The bond lengths and bond angles in the title compound (Fig. 1) are within normal ranges (Allen *et al.*, 1987) and comprable with those observed in similar compounds (Qiu *et al.*, 2006; Yang & Guo, 2006; Yang, 2006). The dihedral angle between the C1—C10 naphthyl ring and C14—C19 benzene ring is 70.1 (2)°. The molecule of the compound adopts a *trans* configuration about the C12═N1 and C13—N2 bonds. In the crystal structure, the molecules are linked into chains along the *c* axis by N—H···O hydrogen bonds (Table 1 and Fig.2).

### Experimental

2-Methoxy-1-naphthaldehyde (0.1 mmol, 18.6 mg) and 3-bromobenzohydrazide (0.1 mmol, 21.5 mg) were dissolved in methanol (10 ml). The mixture was stirred at room temperature for 10 min to give a clear colorless solution. Colourless blocks of (I) were formed by gradual evaporation of the solvent over a week at room temperature (yield 63%).

### Refinement

Atom H2 was located in a difference Fourier map and refined isotropically, with the N—H distance restrained to 0.90 (1) Å. The remaining H atoms were placed in geometrically idealized positions and allowed to ride on their parent atoms, with C—H = 0.93–0.96 Å, and with *U*_iso_(H) = 1.2*U*_eq_(C) and 1.5*U*_eq_(C11).

### Crystal data

**Table:**

C_19_H_15_BrN_2_O_2_	*F*(000) = 776
*M**_r_* = 383.24	*D*_x_ = 1.494 Mg m^−^^3^
Monoclinic, *P*2_1_/*c*	Mo *K*α radiation, λ = 0.71073 Å
Hall symbol: -P 2ybc	Cell parameters from 1085 reflections
*a* = 12.3562 (11) Å	θ = 2.3–24.5°
*b* = 17.0404 (15) Å	µ = 2.43 mm^−^^1^
*c* = 8.6175 (10) Å	*T* = 298 K
β = 110.155 (2)°	Block, colorless
*V* = 1703.3 (3) Å^3^	0.30 × 0.27 × 0.27 mm
*Z* = 4	

### Data collection

**Table:**

Bruker SMART CCD diffractometer	3513 independent reflections
Radiation source: fine-focus sealed tube	1721 reflections with *I* > 2σ(*I*)
graphite	*R*_int_ = 0.080
ω scans	θ_max_ = 26.5°, θ_min_ = 2.4°
Absorption correction: multi-scan (*SADABS*; Sheldrick, 1996)	*h* = −10→15
*T*_min_ = 0.530, *T*_max_ = 0.560	*k* = −21→18
9533 measured reflections	*l* = −10→10

### Refinement

**Table:**

Refinement on *F*^2^	Primary atom site location: structure-invariant direct methods
Least-squares matrix: full	Secondary atom site location: difference Fourier map
*R*[*F*^2^ > 2σ(*F*^2^)] = 0.052	Hydrogen site location: inferred from neighbouring sites
*wR*(*F*^2^) = 0.132	H atoms treated by a mixture of independent and constrained refinement
*S* = 1.00	*w* = 1/[σ^2^(*F*_o_^2^)] where *P* = (*F*_o_^2^ + 2*F*_c_^2^)/3
3513 reflections	(Δ/σ)_max_ < 0.001
221 parameters	Δρ_max_ = 0.30 e Å^−^^3^
1 restraint	Δρ_min_ = −0.36 e Å^−^^3^

### Special details

**Table:**

Geometry. All e.s.d.'s (except the e.s.d. in the dihedral angle between two l.s. planes) are estimated using the full covariance matrix. The cell e.s.d.'s are taken into account individually in the estimation of e.s.d.'s in distances, angles and torsion angles; correlations between e.s.d.'s in cell parameters are only used when they are defined by crystal symmetry. An approximate (isotropic) treatment of cell e.s.d.'s is used for estimating e.s.d.'s involving l.s. planes.
Refinement. Refinement of *F*^2^ against ALL reflections. The weighted *R*-factor *wR* and goodness of fit *S* are based on *F*^2^, conventional *R*-factors *R* are based on *F*, with *F* set to zero for negative *F*^2^. The threshold expression of *F*^2^ > σ(*F*^2^) is used only for calculating *R*-factors(gt) *etc*. and is not relevant to the choice of reflections for refinement. *R*-factors based on *F*^2^ are statistically about twice as large as those based on *F*, and *R*- factors based on ALL data will be even larger.

### Fractional atomic coordinates and isotropic or equivalent isotropic displacement parameters (Å^2^)

**Table:**

	*x*	*y*	*z*	*U*_iso_*/*U*_eq_	
Br1	0.92086 (5)	0.10384 (4)	0.45503 (6)	0.0640 (3)	
N1	0.6290 (3)	0.2794 (2)	0.8906 (4)	0.0378 (10)	
N2	0.7166 (3)	0.2434 (2)	0.8493 (4)	0.0389 (10)	
O1	0.5703 (3)	0.4903 (2)	0.7240 (4)	0.0668 (11)	
O2	0.7770 (3)	0.17262 (19)	1.0857 (4)	0.0534 (9)	
C1	0.4904 (4)	0.3853 (3)	0.8237 (5)	0.0394 (12)	
C2	0.4835 (5)	0.4634 (3)	0.7754 (6)	0.0500 (14)	
C3	0.3937 (6)	0.5126 (3)	0.7840 (7)	0.0658 (17)	
H3	0.3901	0.5649	0.7519	0.079*	
C4	0.3128 (5)	0.4821 (4)	0.8402 (7)	0.0679 (17)	
H4	0.2538	0.5145	0.8460	0.082*	
C5	0.3145 (4)	0.4033 (3)	0.8900 (6)	0.0481 (13)	
C6	0.2266 (5)	0.3738 (4)	0.9432 (7)	0.0690 (17)	
H6	0.1677	0.4069	0.9472	0.083*	
C7	0.2270 (5)	0.2978 (4)	0.9885 (7)	0.0715 (18)	
H7	0.1683	0.2786	1.0223	0.086*	
C8	0.3164 (5)	0.2483 (3)	0.9841 (7)	0.0618 (15)	
H8	0.3172	0.1963	1.0170	0.074*	
C9	0.4018 (4)	0.2751 (3)	0.9328 (6)	0.0490 (14)	
H9	0.4596	0.2407	0.9302	0.059*	
C10	0.4055 (4)	0.3543 (3)	0.8829 (6)	0.0417 (12)	
C11	0.5644 (6)	0.5702 (3)	0.6675 (7)	0.083 (2)	
H11A	0.4904	0.5796	0.5845	0.124*	
H11B	0.6239	0.5792	0.6215	0.124*	
H11C	0.5749	0.6052	0.7589	0.124*	
C12	0.5855 (4)	0.3402 (3)	0.8040 (6)	0.0425 (12)	
H12	0.6159	0.3564	0.7245	0.051*	
C13	0.7872 (4)	0.1907 (3)	0.9535 (5)	0.0364 (11)	
C14	0.8782 (4)	0.1572 (3)	0.8967 (6)	0.0338 (11)	
C15	0.8636 (4)	0.1497 (3)	0.7312 (6)	0.0372 (12)	
H15	0.7964	0.1678	0.6512	0.045*	
C16	0.9484 (4)	0.1153 (3)	0.6847 (6)	0.0437 (12)	
C17	1.0496 (5)	0.0893 (3)	0.8001 (7)	0.0605 (16)	
H17	1.1066	0.0666	0.7671	0.073*	
C18	1.0652 (5)	0.0975 (4)	0.9653 (7)	0.0758 (19)	
H18	1.1335	0.0806	1.0446	0.091*	
C19	0.9793 (5)	0.1309 (3)	1.0143 (6)	0.0548 (15)	
H19	0.9898	0.1356	1.1261	0.066*	
H2	0.731 (4)	0.266 (3)	0.764 (4)	0.066*	

### Atomic displacement parameters (Å^2^)

**Table:**

	*U*^11^	*U*^22^	*U*^33^	*U*^12^	*U*^13^	*U*^23^
Br1	0.0692 (4)	0.0859 (5)	0.0489 (3)	0.0221 (3)	0.0355 (3)	0.0008 (3)
N1	0.042 (2)	0.045 (3)	0.033 (2)	0.006 (2)	0.0207 (19)	−0.0008 (19)
N2	0.043 (3)	0.046 (3)	0.037 (2)	0.007 (2)	0.025 (2)	0.006 (2)
O1	0.089 (3)	0.047 (2)	0.068 (3)	−0.006 (2)	0.032 (2)	0.0087 (19)
O2	0.073 (2)	0.057 (2)	0.041 (2)	0.0126 (19)	0.0327 (19)	0.0088 (18)
C1	0.045 (3)	0.037 (3)	0.033 (3)	0.009 (3)	0.010 (2)	−0.003 (2)
C2	0.063 (4)	0.049 (4)	0.037 (3)	0.008 (3)	0.015 (3)	−0.003 (3)
C3	0.083 (5)	0.041 (4)	0.064 (4)	0.013 (4)	0.012 (4)	0.001 (3)
C4	0.058 (4)	0.069 (5)	0.071 (4)	0.026 (3)	0.015 (3)	−0.002 (3)
C5	0.048 (3)	0.051 (4)	0.040 (3)	0.009 (3)	0.010 (3)	0.000 (3)
C6	0.051 (4)	0.079 (5)	0.077 (4)	0.015 (3)	0.022 (3)	−0.007 (4)
C7	0.050 (4)	0.102 (6)	0.072 (4)	−0.001 (4)	0.032 (3)	0.001 (4)
C8	0.059 (4)	0.063 (4)	0.068 (4)	0.006 (3)	0.027 (3)	−0.002 (3)
C9	0.049 (4)	0.052 (4)	0.051 (3)	0.012 (3)	0.024 (3)	0.004 (3)
C10	0.041 (3)	0.046 (3)	0.037 (3)	0.011 (3)	0.011 (2)	−0.007 (2)
C11	0.127 (6)	0.051 (4)	0.071 (4)	−0.024 (4)	0.035 (4)	0.005 (3)
C12	0.052 (3)	0.046 (3)	0.032 (3)	0.003 (3)	0.018 (2)	−0.001 (2)
C13	0.047 (3)	0.038 (3)	0.029 (3)	0.000 (2)	0.018 (2)	0.000 (2)
C14	0.035 (3)	0.034 (3)	0.036 (3)	0.001 (2)	0.017 (2)	0.000 (2)
C15	0.034 (3)	0.036 (3)	0.043 (3)	0.001 (2)	0.016 (2)	0.001 (2)
C16	0.048 (3)	0.048 (3)	0.042 (3)	0.007 (3)	0.025 (3)	0.001 (2)
C17	0.046 (3)	0.079 (4)	0.061 (4)	0.018 (3)	0.025 (3)	0.001 (3)
C18	0.053 (4)	0.114 (6)	0.054 (4)	0.040 (4)	0.011 (3)	0.005 (4)
C19	0.062 (4)	0.069 (4)	0.034 (3)	0.011 (3)	0.017 (3)	0.001 (3)

### Geometric parameters (Å, °)

**Table:**

Br1—C16	1.899 (5)	C7—H7	0.9300
N1—C12	1.282 (5)	C8—C9	1.356 (6)
N1—N2	1.392 (5)	C8—H8	0.9300
N2—C13	1.354 (6)	C9—C10	1.423 (6)
N2—H2	0.898 (10)	C9—H9	0.9300
O1—C2	1.372 (6)	C11—H11A	0.9600
O1—C11	1.439 (6)	C11—H11B	0.9600
O2—C13	1.228 (5)	C11—H11C	0.9600
C1—C2	1.389 (7)	C12—H12	0.9300
C1—C10	1.417 (6)	C13—C14	1.487 (6)
C1—C12	1.462 (6)	C14—C15	1.380 (6)
C2—C3	1.413 (7)	C14—C19	1.384 (6)
C3—C4	1.355 (7)	C15—C16	1.375 (6)
C3—H3	0.9300	C15—H15	0.9300
C4—C5	1.408 (7)	C16—C17	1.377 (7)
C4—H4	0.9300	C17—C18	1.376 (7)
C5—C6	1.410 (7)	C17—H17	0.9300
C5—C10	1.418 (6)	C18—C19	1.393 (7)
C6—C7	1.351 (8)	C18—H18	0.9300
C6—H6	0.9300	C19—H19	0.9300
C7—C8	1.401 (7)		
			
C12—N1—N2	114.5 (4)	C1—C10—C9	124.6 (4)
C13—N2—N1	120.2 (4)	C5—C10—C9	115.9 (5)
C13—N2—H2	124 (3)	O1—C11—H11A	109.5
N1—N2—H2	114 (3)	O1—C11—H11B	109.5
C2—O1—C11	118.1 (5)	H11A—C11—H11B	109.5
C2—C1—C10	119.3 (5)	O1—C11—H11C	109.5
C2—C1—C12	116.0 (5)	H11A—C11—H11C	109.5
C10—C1—C12	124.7 (4)	H11B—C11—H11C	109.5
O1—C2—C1	116.3 (5)	N1—C12—C1	123.4 (4)
O1—C2—C3	122.3 (5)	N1—C12—H12	118.3
C1—C2—C3	121.4 (5)	C1—C12—H12	118.3
C4—C3—C2	118.7 (6)	O2—C13—N2	122.2 (4)
C4—C3—H3	120.6	O2—C13—C14	122.6 (4)
C2—C3—H3	120.6	N2—C13—C14	115.1 (4)
C3—C4—C5	122.7 (5)	C15—C14—C19	119.4 (4)
C3—C4—H4	118.7	C15—C14—C13	122.1 (4)
C5—C4—H4	118.7	C19—C14—C13	118.5 (4)
C4—C5—C6	120.4 (6)	C16—C15—C14	119.9 (4)
C4—C5—C10	118.5 (5)	C16—C15—H15	120.0
C6—C5—C10	121.1 (5)	C14—C15—H15	120.0
C7—C6—C5	120.5 (6)	C15—C16—C17	121.4 (4)
C7—C6—H6	119.7	C15—C16—Br1	117.8 (4)
C5—C6—H6	119.7	C17—C16—Br1	120.8 (4)
C6—C7—C8	119.6 (6)	C18—C17—C16	118.9 (5)
C6—C7—H7	120.2	C18—C17—H17	120.5
C8—C7—H7	120.2	C16—C17—H17	120.5
C9—C8—C7	121.0 (6)	C17—C18—C19	120.4 (5)
C9—C8—H8	119.5	C17—C18—H18	119.8
C7—C8—H8	119.5	C19—C18—H18	119.8
C8—C9—C10	121.8 (5)	C14—C19—C18	120.0 (5)
C8—C9—H9	119.1	C14—C19—H19	120.0
C10—C9—H9	119.1	C18—C19—H19	120.0
C1—C10—C5	119.5 (5)		

### Hydrogen-bond geometry (Å, °)

**Table:**

*D*—H···*A*	*D*—H	H···*A*	*D*···*A*	*D*—H···*A*
N2—H2···O2^i^	0.90 (1)	2.10 (1)	2.989 (5)	174 (5)

Symmetry codes: (i) *x*, −*y*+1/2, *z*−1/2.
